# A qualitative exploration of the impact of a hospital electronic prescribing and medicines administration (HEPMA) protocol on junior doctor confidence and competence to prescribe end-of-life care medicines

**DOI:** 10.1007/s11096-024-01789-9

**Published:** 2024-08-07

**Authors:** Ewan McLean, Amanda McLean, Marion Bennie

**Affiliations:** 1 Western General Hospital, NHS Lothian, Edinburgh, UK; 2 Strathclyde Institute of Pharmacy & Biomedical Sciences (SIPBS), University of Strathclyde, Glasgow, UK

**Keywords:** Computerized physician order entry, Hospital electronic prescribing and medicines administration, Palliative care, Prescribing

## Abstract

**Background:**

With hospital electronic prescribing and medicines administration (HEPMA) systems now in widespread use across hospital inpatient clinical services, work is underway to measure the benefits of HEPMA on healthcare systems and patient care. HEPMA functionality enables users to prescribe medicines by ‘bundle’ or ‘protocol’. Although it is assumed that this is a significant system benefit, there are few qualitative studies supporting this conclusion.

**Aim:**

To explore the impact of an electronic anticipatory care medicines protocol on junior doctor perceptions of their confidence and competence to prescribe opioids and midazolam for patients at the end of life.

**Method:**

Between May and August 2022, one-to-one semi-structured interviews were conducted at a 570-bed District General Hospital with junior doctors who had experience of prescribing on both HEPMA and paper-based systems. Audio recordings of the interviews were transcribed verbatim and underwent thematic analysis.

**Results:**

Ten junior doctors participated (median age 23 years). Analysis generated five main themes that described perceptions and attitudes towards confidence and competence. These were prescribing safety benefits; information technology infrastructure, interoperability and system design concerns; clinical knowledge and training needs; cultural and social factors and risks of automation in prescribing.

**Conclusion:**

This study suggests that junior doctors experienced an overall increase in their confidence and perceived competence to prescribe anticipatory medicines post-implementation of a HEPMA protocol. Further studies are required to detail the impact of HEPMA/CPOE protocols on clinical practice.

**Supplementary Information:**

The online version contains supplementary material available at 10.1007/s11096-024-01789-9.

## Impact statements

- Implementation of protocol-focussed prescribing on HEPMA/CPOE systems can improve perceived prescribing safety and efficiency, though risks such as automation hazards may introduce clinical risk.
- Positive and negative factors contributing to perceptions of the HEPMA/CPOE anticipatory protocol are described in this study and provide an understanding of the opportunities and challenges offered by protocol-based prescribing.
- Future research should evaluate a wider range of HEPMA/CPOE protocols across multiple centres to develop understanding of the impact of these protocols on clinical practice.

## Introduction

Hospital electronic prescribing and medicines administration (HEPMA) [or Computerized Physician Order Entry (CPOE)] systems have been implemented globally across acute hospital sites, primary care, general practice and residential care facilities. HEPMA systems are designed to replace paper-based processes for medicines prescribing and administration [1]. The overall aim is to improve clinical care for patients. Advantages of electronic prescribing systems are outlined in some published literature [2–4]. Some advantages are improved legibility and quality of prescribing, improved clinical decision support and vast data availability on medicines prescribing and administration. Limitations to HEPMA are reported in other studies. These include accessibility concerns, issues with system speed due to interface design and lack of standardisation upon implementation [5]. There are few published studies with a focus on HEPMA/CPOE user perceptions of their abilities to practice and the system impact on user capabilities. Published work describes a need for more research to better outline the impact of these systems on healthcare professionals practice, clinical workflow and safety [6, 7].

In the United Kingdom (UK), junior doctors are widely recognised as the majority prescribers in the acute hospital setting. A small number of studies highlight a specific area of risk, the prescribing of end-of-life care (anticipatory) medicines including opioids and midazolam. Paper-prescribing focussed studies assessed the prescribing of opioids and midazolam and have shown common errors in the prescribing of opioids and midazolam [8]. One study outlines the common errors in the prescribing of anticipatory medicines, which include inappropriate and suboptimal doses, incorrect dosing frequency and a lack of awareness of anticipatory prescribing guidelines amongst medical staff [9]. Another qualitative study has shown that junior doctors feel under-equipped to prescribe for dying patients. This research has suggested that revised medical education is required to address this need [10]. A further small study illustrates the merit of multidisciplinary teaching and training on the confidence of doctors to prescribing opioids safely and effectively [11].

In Scotland, HEPMA implementation is a key policy goal with estimated cost of £24M [12]. Local and national work is underway to describe system benefits. Given significant implementation costs and health service financial pressures, it is essential that the benefits of HEPMA are measured by local and national benefits realisation work [13]. As HEPMA becomes integrated within healthcare services, maximisation of national benefits realisation and shared learning is key.

One area of exploration is the adoption of electronic prescribing protocols. Protocols are sets of medicines grouped together for prescribing in a single action. It is suggested that this a potential key HEPMA/CPOE benefit in terms of usability, efficiency, and safety [14]. At an NHS Scotland Hospital, a HEPMA ‘Anticipatory Medicines Protocol’ has been developed and implemented across all clinical areas. This protocol reflects local guidelines for ‘Anticipatory Prescribing’ [15] and combines anticipatory (‘just-in-case’) medicines within a single selection for prescribers. This enables prescribers to generate a ‘set’ of pre-programmed medicines and doses that can be amended as required by the prescriber. The result is a standardised prescription for end-of-life medicines for inpatients and on discharge.

The research team has been unable to find any published studies examining the impact of electronic prescribing protocol on the confidence or competence of doctors in prescribing opioids and midazolam for patients at the end of life.

### Aim

This study aimed to explore the impact of an electronic anticipatory care medicines protocol on junior doctor perceptions of their confidence and competence to prescribe opioids and midazolam for patients at the end of life.

### Ethics approval

The University of Strathclyde Ethics Officer approved this study on 6th April 2022 (approval number EA21-15) following completion of the university research approval form [16]. As this study used prescribing and patient data, local health authority and senior management approval was obtained on 27th January 2022. Local research team approval was also obtained.

## Method

### Design

The study was qualitative, using semi-structured one–one interviews. Focus groups were deemed unpractical due to clinical service needs. The study was undertaken at a District General Hospital (DGH) within NHS Lothian, Scotland, the second largest regional health authority in Scotland serving a population of approximately 850,000 people [17]. The DGH has a bed capacity of circa 570 [18]. Phased implementation of the HEPMA system took place between March 2021 and June 2021 and the HEPMA Anticipatory Medicines Protocol was implemented in April 2021.

A target 10–14 participants was set for recruitment based on site staffing. It is recognised that 9–17 participants are required to meet 90% data saturation [19, 20]. The intended goal of the sample was to provide experiences from junior prescribers whilst also providing experience from multiple clinical specialities.

Participant inclusion criteria were foundation year doctors (prescribers) practicing within first 5 years post registration qualification, who had experience of using the DGH HEPMA system and anticipatory medicines prescribing protocol were eligible for inclusion in this study. This was to ensure the impact on junior doctor staffing was minimised. Exclusion criteria were non-medical prescribers, prescribers who have completed formal training in palliative care (e.g. diploma, Master of Science (MSc)) and prescribers with more than five years post registration experience. The Consolidated Criteria for Reporting Qualitative Studies (COREQ) checklist was used to ensure the quality of reporting of qualitative research.

### Recruitment

A participant information leaflet was communicated by internal email to DGH senior clinicians to disseminate to junior staff. Interested participants were invited to contact the Lead Investigator by email or telephone. Further participants were identified via the snowball sampling method. Snowball sampling is a method in which research participants are asked to assist researchers in identifying other potential participants [21]. Snowball sampling—rather than purposive sampling—was select to ensure participants were recruited quickly given the short timeframe for useful data collection. After registering interest prospective participants were issued with consent forms which were completed and returned by email or in person. Participation in the study was not incentivised.

### Data collection and analysis

A semi-structured interview schedule was developed by the lead investigator in conjunction with an experienced, trained pharmacist researcher (AM) (Appendix 1) based at the same hospital. Credibility of the interview schedule was reviewed by AM. The interview schedule was piloted on a junior doctor with recent experience of both paper-based and electronic prescribing. Following the pilot interview phase, the interview schedule was modified to remove quantitative data collection using a likert scale. Reasons for this are described in the strengths and weaknesses of the study. Pilot interview data were excluded from the study.

All interviews were completed by the lead investigator, EMcL, an experienced UK hospital pharmacist. EMcL conducted interviews privately, in person, face-to-face, between May and August 2022 with participants coded (P1–10). Participants were reassured regarding anonymity. A digital recorder was utilised to record audio of the interviews. The digital recorder and audio files were stored securely to maintain participant confidentiality. Each audio recording was transcribed verbatim by the Lead Investigator using Microsoft Office Word software. AM validated 20% of the transcriptions to ensure accuracy. Following transcription and validation, all recordings were deleted. All transcript copies, written consent forms and demographic information were stored securely in a locked file.

Transcripts were uploaded to qualitative data management software NVivo^©^ (QSR International Ltd). Framework (thematic) analysis method was completed according to Braun and Clarke’s recommended six phases [22]. Triangulation took place as EMcL and AM themed the transcripts independently. Discussion regarding themes was conducted between the researchers; data were then grouped as themes with sub-themes identified within these themes.

EMcL (MPharm) and AM (MPhil, DProf) are male and female pharmacists who work at the same hospital site, with 7 and 25 years experience respectively. AM, but not EMCL, had experience in qualitative research.

## Results

Overall, 10 individuals were recruited and attended for interview. Duration of interview ranged from 21 to 32 min. The aim of recruiting to data saturation was not achieved due to clinical pressures and demands on the time of junior doctors working in front-line secondary care. Data saturation was not achieved as new data was output until the tenth interview was concluded and analysed. The characteristics of the 10 junior doctors are presented in Table 1. Most participants were female (n = 7) and median age was 23 years. Other characteristics showed similarities in the professional experience of the participants. All 10 participants had significant experience of both HEPMA and paper-based prescribing. All 10 participants described some experience of protocol-based prescribing of anticipatory medicines as per the recruitment criteria. Five key themes and associated subthemes identified from the interviews are summarised in Table 2.

**Table 1:** Characteristics of participating doctors

Variables	Eligible participants at FY1 level	Eligible participants at FY2 level
Age (Years)	P1, P2, P4, P5, P6, P7, P9, P10	P3, P8
Median 23	Surgery-based participants	Surgery-based participant
IQR 22-23	P1, P2, P4, P5, P7	P8
Gender, n	Medicine based patricipants	Oncology-based participant
Female, 7	P6, P9, P10	P3
Male, 3

**Table 2:** Themes identified from interviews

Interview schedule theme	Subtheme
Prescribing safety benefits	Standardisation, security and trust
	Reduction in errors
	Efficiency
IT infrastructure, interoperability and system design factors	HEPMA system access challenges
	Concerns over system design
Clinical knowledge and training	Clinical understanding
	Training needs
Cultural and social factors	
Risks of automation in electronic prescribing	

### Theme 1: Prescribing safety benefits

#### Subtheme: Standardisation, security and trust

Participants noted that the electronic anticipatory care protocol provided them with feelings of safety and security as they prescribed. Some noted this was due to inbuilt system functionality, such as interaction checking, whilst others felt this was due to the standardisation provided by prescribing protocols such as the anticipatory care protocol. A sense of trust in the HEPMA system was recognised across some participants.

*‘Having used the protocol … I feel there is a safeguard there and that provides me with a little bit of reassurance that there is a check going on. It tells you that something is looking over you and making you second check yourself which I think is helpful’ P1.*

*‘Because the protocol is based on approved guidelines and the information is pre-populated, I don’t have to do too much. When I prescribe a protocol, this is when I feel most secure as a relatively new prescriber. I can trust it’ P3.*

#### Subtheme: Reduction in prescribing errors

The majority of participants felt they were less likely to make a mistake when prescribing protocols on the HEPMA system, though it was felt that protocols could not completely remove risk when prescribing.

*‘I absolutely feel there is less room for error, you know. If you prescribe by protocol, all elements are included. So the chance of you missing something is next to zero, unless the person creating the protocol has made a mistake.’ P7.*

*‘I do remember prescribing the anticipatory medicines via the intravenous route (IV) when I first prescribed them …. The protocol would have corrected that if I had been aware of it.’ P5*

*‘I think without the protocol, some of the medicines would be missed.’ P4*

#### Subtheme: Time saving and efficiency

Use of the anticipatory care protocol enabled participants to work more efficiently in the clinical areas. Time-saving benefits were reported by one prescriber.

*‘There was a patient who needed the ‘just-in-case’ (anticipatory) medicines prescribed quickly … I felt a bit overwhelmed because it was on a weekend and there weren’t many doctors around, but then one of the registrars reminded me that the protocol was there. It took the pressure off and I was able to prescribe … quickly’. P4.*

Another participant made comparison between the efficiency of prescribing anticipatory medicines on paper versus HEPMA, noting similarities and the availability of the protocol on paper.

*‘On paper the protocol was there too which was good. The problem was that the paper proforma was a separate sheet of paper and that was in amongst paper notes .., so it could be lost or difficult to find..I remember delays to treatment due to trying to find it. P10’*

### Theme 2: IT infrastructure, interoperability and system design factors

#### Subtheme: Difficulties in accessing the HEPMA system

Most participants reported experiencing difficulties in using the HEPMA system due to system slowness caused by network issues. These difficulties appeared to reduce user confidence in the system to be able to prescribe quickly enough for the patient in front of them.

*‘HEPMA is slow… I often have to wait several minutes to complete an action. For example, I was asked by my consultant to prescribe ACPs (anticipatory medicines) on a ward round. By the time I … loaded HEPMA, we had already moved on to the next patient. This feels risky to me.’ P9.*

#### Subtheme: Concerns over system design

Anxieties over system design and reliance on accurate protocol design is described by the data. It is evident that prescribers have concerns that they are following the system design without due clinical consideration.

*‘I prescribed the (anticipatory) bundle for a patient recently. Levomepromazine can be given more regularly for terminal agitation – I mean, the bundle states a maximum of 2 hourly administrations but the guidelines say maximum 1 hourly. I worry that we have to rely on the design of the bundle being correct. Because we will follow the design’. P2.*

### Theme 3: Clinical knowledge, understanding and training needs

#### Subtheme: Unawareness of local protocols

Gaps in clinical understanding were identified during interviews. One participant was unaware of local guidance which forms the basis for the HEPMA protocol.

*‘When I look for guidance (about anticipatory prescribing), I look to the BNF (British National Formulary). Also, the palliative care team are a really helpful resource and support me well and pharmacy too.’ P1.*

Data demonstrated perceived benefits in junior prescriber adherence to nationally approved guidance. It was acknowledged that there was risk of inappropriate drug selection but this was mitigated by the HEPMA anticipatory protocol.

*‘I think without the protocol I would’ve prescribed any antiemetic, say cyclizine, not knowing that the broader spectrum option is levomepromazine. So that guidance is really helpful and means my prescribing is as effective as it can be’ P7.*

#### Subtheme: Training needs

Need for further training in palliative care and associated prescribing was described in the interviews. Some participants felt they would have benefited from additional sessions on anticipatory prescribing as undergraduate trainees and further sessions as newly qualified foundation doctors. The negative impact of COVID-19 on undergraduate training was also noted.

*‘…. I don’t feel that the training before I came into FY (foundation year) equipped me to prescribe ACPs effectively. I wasn’t confident in prescribing the medicines … perhaps there is a training need there’ P2.*

*‘’From medical school, I definitely knew where the (palliative care) guidelines were but most of my palliative care training was online due to COVID so maybe I was at a disadvantage’ P10*

### Theme 4: Cultural and social factors

Several participants described feelings of anxiety when prescribing anticipatory medicines, particularly in the early months of practicing as a doctor. Others felt confident and competent prior to implementation of the HEPMA protocol.

*‘When I first started using HEPMA, I didn’t feel confident in prescribing anticipatory medicines. They are quite potent. I check and I double check that I am doing the right thing because these medicines have the potential to be toxic to the patient’. P2.*

*‘I didn’t mind prescribing medicines for EOLC (end of life care) patients. I felt comfortable prescribing them, the protocol helped with that comfort I suppose.’ P3*

Data showed that a number of the junior doctors who participated were aware of public and media perception around the prescribing of opioids and midazolam and that these cultural perceptions impacted their confidence and competence to prescribe these medicines.

*‘Even prescribing a low dose opioid as an FY1 … source of anxiety to me as it is a controlled substance. There is this feeling of there being a cultural or social impact to prescribing these medicines … I did feel some worry’ P8.*

### Theme 5: Risks of automation in electronic prescribing

Consequences of electronic prescribing protocols were considered by participants. Participants proposed potential risks such as lack of thought and clinical consideration when prescribing by protocol. Others noted the dangers of click fatigue.

*‘I would worry about associated thoughtlessness when prescribing protocols … a clinical decision needs to be made about treatment versus no treatment. Perhaps the protocols encourage me to treat, it’s just something I’ve thought about.’ P6.*

*‘There’s a potential danger – depending on how you teach people to use the system. Avoid HEPMA system training being a click exercise and pair it with clinical and it reduces the risk. P9*

Automation concerns were highlighted by few participants but evidently were significant in terms of their trust in the system and confidence to prescribe by protocol.

## Discussion

### Statement of key findings

Our study found that an overall increase in confidence and competence to prescribe end of life care medicines was described by participants after the HEPMA/CPOE protocol was implemented. Multiple factors contributed to participant perceptions and attitudes towards their ability to practice. These included: acknowledgement of prescribing safety benefits, with specific focus on standardisation, error reduction and efficiency gains; concerns over IT infrastructure, interoperability and system design; gaps in clinical knowledge and training; the impact of cultural and social factors on prescribing of opioids and midazolam and potential risks of automation in electronic prescribing.

### Interpretation

Participants highlighted positive effects of the HEPMA anticipatory protocol on feelings of safety and security when prescribing for patients, as well as reduced likelihood that they would make an error. Previous studies suggest improved user confidence is observed when CPOE systems provide standardised instructions [23, 24]. One Australian study concluded that CPOE protocols may improve clinical practice; these findings appear to align with those of our study, as participants consistently reported perceptions of reduced errors and improved efficiency when prescribing [25]. Time saving benefits were noted by several participants. The protocol was generally felt to improve the quickness at which medicines were prescribed whilst maintaining standardisation.

Effects of IT infrastructure and reliability of IT systems was a focus for several participants in their responses. Efficiency provided by the HEPMA anticipatory protocol was hampered by IT system slowness and failure, which appeared to reduce system confidence (and by extension reduce confidence in protocol-based prescribing). Responses from our study show that electronic systems require robust platforms and interfaces between co-dependent systems. Deficiencies in IT infrastructure will negatively impact the ability of the end-user (prescriber) to practice with confidence. These findings are substantiated by existing literature [26–28].

Most participants highlighted the importance of education and training on their confidence and competence to prescribing anticipatory medicines. There were notable feelings of worry around prescribing opioids and midazolam which are perceived as specialised medicines that carry risk. Some junior doctors believed that despite being majority system users, more clinical and system training would have improved their confidence and competence to prescribe effectively; previous international studies have reported decreased errors following improved prescriber training [29, 30].

In most participants responses, feelings of worry and stress associated with the prescribing opioids and midazolam were evident. The need to overcome cultural and social stigma attached to opioid prescribing was also prevalent in the data. Junior doctors showed awareness of public and media perception of the use of opioids and midazolam, and this negatively impacted their confidence and competence to prescribing end of life care medicines. No other studies appear to have reported this outcome.

Significantly, participants provided insight to the risks of automation associated with electronic prescribing protocols and system over-reliance. It was clear that protocol users were concerned that prescribers may be more likely to prescribe a protocol (and all drugs within that protocol) to their patient without full clinical consideration. One Australian study highlighted similar risks in over-reliance on HEPMA/CPOE decision support for interactions [31]. This unintended consequence of protocol implementation carries a risk to patient safety; the research team therefore recommend full risk assessment of each protocol prior to implementation, including robust clinical system testing in consultation with specialist clinical teams.

### Strengths and weaknesses

To the best of our knowledge, this is the first study to have explored the perceptions and attitudes of junior doctors on the impact of an electronic prescribing protocol on their confidence and competence to practice. All participants in this study had recent experience of both HEPMA/CPOE system prescribing and paper-based prescribing. A strength of this study was the timing of data collection which allowed for comparison of paper and HEPMA prescribing, as HEPMA/CPOE will phase out paper systems in the coming years.

There were some limitations to our study. The intended interview schedule included data collection aimed at quantitative data collection using a likert scale. However, due to low recruitment the sample size was not seen to be adequate. To mitigate for this weakness, the research team attempted to recruit more participants, but this was not possible due to clinical service demand. The interviewer (EMcL) was known to several participants, through multidisciplinary working in clinical practice, which may have introduced some bias to participant responses. Further, the study was conducted in only one regional health board. This health board uses one HEPMA/CPOE system, therefore it is unclear if highlighted concerns would arise in other systems and our findings may lack generalisability.

### Further research

This small-scale study demonstrates some benefits of HEPMA/CPOE protocols on junior doctor confidence and competence to prescribe specialised medicines, such as opioids and midazolam, for patients at the end of life. More research is needed to determine whether findings of improved safety and security are transferable across greater numbers of users, protocol-types, alternative EPMA/CPOE systems and health services. Larger studies are necessary to evaluate the impact of protocol-focused prescribing via HEPMA/CPOE systems on prescribing and administration errors. Our small-scale study detailed risks of protocol-based prescribing in the inpatient setting, and future studies should give focus to outpatient clinical areas, where HEPMA/CPOE systems are embedded internationally. A prospective study could involve a large-scale multi-centre mixed-method study with wider prescriber participation (i.e. inclusion of senior doctors, nurse and pharmacist prescribers). Prospective research should also assess the risks of protocol-based prescribing; our study suggests that patients may be prescribed unnecessary medications via HEPMA/CPOE protocols due to automation.

## Conclusion

Findings from one-to-one interviews indicated that prescribers experienced improved confidence and competence to prescribe end-of-life care medicines when using a HEPMA/CPOE protocol. In this qualitative study several complex factors contributing to perceptions of confidence and competence were identified following HEPMA/CPOE protocol implementation. This research suggests HEPMA/CPOE protocols can standardise practice and cultivate feelings of safety and trust from users. Wider research is required to explore the impacts on practice associated with protocol-based prescribing, such as automation-related clinical risk.

## Supplementary Information

Below is the link to the electronic supplementary material.

Supplementary file1 (DOCX 15 KB)
