# Supplementary material for: A qualitative exploration of the impact of a hospital electronic prescribing and medicines administration (HEPMA) protocol on junior doctor confidence and competence to prescribe end-of-life care medicines
Source: Int J Clin Pharm. 2024 Aug 7;46(6):1445–52. doi: 10.1007/s11096-024-01789-9 (PMC11576621; doi:10.1007/s11096-024-01789-9)
Supplement: Supplementary file 1 — Supplementary file1 (DOCX 15 KB) [file 11096_2024_1789_MOESM1_ESM.docx]

Appendix 1

**Supplementary Appendix 1: Semi-Structure Interview Schedule for junior prescribers with experience of the HEPMA/CPOE anticipatory medicines protocol**

1. Demographics:

- M/F
- Job Role
- Age

2. Qualitative interview introduction

- Length (max 30 mins)

Primary goal: To understand the impact of the HEPMA Anticipatory Medicines Protocol on junior doctor confidence and competence to prescribe opioids and midazolam for patients at the end of life

i.e. to build understanding of your perceptions around the HEPMA system (specifically the ACP protocol) and its impact on your confidence and competence to prescribe opioids and midazolam

3. Background information

- Tell me about your experience so far as a doctor, rotational jobs, sites worked on and career plans

4. Can you tell me about your experience in using the HEPMA system to date?

- Previous job use
- Current use
- General positives/negatives

5. Can you tell me about your experience in prescribing medicines for patients at the end of life (e.g. opioids/midazolam)

PROMPT: Have you had to prescribe anticipatory medicines?

PROMPT: Describe the challenges of prescribing anticipatory medicines, if any?

PROMPT: How have you prescribed these? (e.g. individually or as per protocol)

6. Can you tell me about the difference, in your opinion, between prescribing opioids and midazolam on paper medication charts versus HEPMA?

PROMPT: Clinical challenges associated with paper, technical challenges of same

PROMPT: Describe your confidence to practice on paper

PROMPT: Describe your competence to practice on paper

PROMPT: Describe your confidence to practice on HEPMA

PROMPT: Described your competence to practice on HEPMA

7*. Since the introduction of HEPMA, do you feel more or less confident to prescribe opioids and midazolam for patients at the end of life?

8*. Since the introduction of HEPMA, do you feel more or less competent to prescribe opioids and midazolam for patients at the end of life?

9. What are, in your view, the strengths of the HEPMA ACP protocol?

PROMPT: Does it make prescribing anticipatory medicines quicker?

PROMPT: Does it make prescribing anticipatory medicines clearer?

10. What, in your view, could be improved about the HEPMA ACP protocol?

PROMPT: Opportunity to feedback any other relevant experiences regarding the HEPMA anticipatory protocol

*NOTE BELOW: MP807 COREQ CHECKLIST ATTACHED WITHIN SUBMISSION DOCUMENT (REQUIRED FOR IJCP)*
